# Pharmacokinetics of Ginsenoside Rb1, Rg3, Rk1, Rg5, F2, and Compound K from Red Ginseng Extract in Healthy Korean Volunteers

**DOI:** 10.1155/2022/8427519

**Published:** 2022-01-24

**Authors:** Hyung Joong Kim, Tae Kyu Oh, Yoon Hee Kim, Jaesun Lee, Joo Myung Moon, Yong Sun Park, Chang Min Sung

**Affiliations:** ^1^R&D Center, BTC Corporation, No. 703, Technology Development Centre, Gyeonggi Technopark, 705 Haean-Ro, Sangnok-Gu, Ansan-Si, Gyeonggi-Do 15588, Republic of Korea; ^2^R&D Center, Bio-Medieng Co., Ltd, No. 805, 137 Sagimakgol-Ro, Jungwon-Gu, Seongnam-Si, Gyeonggi-Do, Republic of Korea; ^3^Bestian Clinical Trial Center, 191 Osongsaengmyeong 1-Ro, Osong-Eup, Heungdeok-Gu, Cheongju-Si, Chungcheongbuk-Do 361-951 28161, Republic of Korea

## Abstract

Individual differences in ginsenoside pharmacokinetics following ginseng administration in humans are still unclear. We aimed to investigate the pharmacokinetic properties of various ginsenosides, including Rb1, Rg3, Rg5, Rk1, F2, and compound K (CK), after a single oral administration of red ginseng (RG) and bioconverted red ginseng extract (BRG). This was a randomized, open-label, single-dose, single-sequence crossover study with washout every 1 week, and 14 healthy Korean men were enrolled. All subjects were equally assigned to two groups and given RG or BRG capsules. The pharmacokinetic parameters of ginsenosides were measured from the plasma drug concentration–time curve of individual subjects. Ginsenosides Rg3, Rk1 + Rg5, F2, and CK in the BRG group showed a higher *C*_max_, AUC_(0–t)_, and AUC_(0–∞)_ and shorter *T*_max_ (for CK) than those in the RG group. These results suggest that BRG may lead to a higher absorption rate of bioactive ginsenosides. This study provides valuable information on the pharmacokinetics of various bioactive ginsenosides, which is needed to enhance the therapeutic efficacy and pharmacological activity of ginseng.

## 1. Introduction

**Figure 1:**
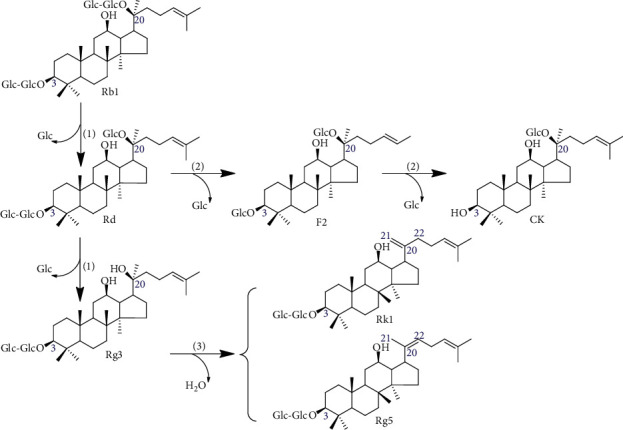
The chemical structure and transformation pathway of protopanaxadiol-type ginsenosides; (1) elimination of terminal glucose residue at C-20; (2) elimination of terminal glucoside residue at C-3; (3) dehydration at C-20.

*Panax ginseng* C.A. Meyer is a beneficial herb that has been consumed for a long time by people in East Asian countries [1, 2]. Consumption of ginseng-based products, such as red ginseng (RG), as a health food has rapidly increased worldwide in recent years [3, 4]. Generally, saponins in ginseng, called ginsenosides, are regarded as the major active components of ginseng with multiple pharmacological activities. Ginseng saponins are structurally classified into protopanaxadiol (PPD), protopanaxatriol, and oleanane saponins according to the position of sugar molecules in their triterpenoid aglycone [5–8]. Furthermore, ginsenosides are grouped as high- and low-molecule ginsenosides based on the number of attached sugar moieties [8]. Among these, Rb1 (a hydrophilic ginsenoside) is categorized as a ginsenoside of PPD type and is the most abundant ginsenoside in ginseng. It is the parent compound of the less hydrophilic ginsenosides Rd, Rg3, Rk1, Rg5, F2, and compound K (CK) [9, 10] (Figure 1). These PPD-type ginsenosides are known to impart various pharmacological effects, such as anti-inflammatory, antitumor, anticancer, antioxidant, antidiabetic, antiallergic, and neuroprotective effects [5, 11–19].

Following oral administration of ginseng or RG, the hydrophilic ginsenosides are converted into hydrophobic ginsenosides by gut microbiota [10, 19]. These hydrophobic ginsenosides (Rg3, Rk1, Rg5, F2, and CK) are deglycosylated by the hydrolysis of hydrophilic ginsenosides [9]. Some studies have reported that because hydrophobic ginsenosides are easier to absorb and easily bind to cell membranes, they have stronger biological activities than hydrophilic ginsenosides [10–13]. However, the ability of an individual to convert these ginsenosides varies widely depending on conditions such as diet, health, and stress [20].

Various methods have been developed to convert hydrophilic ginsenosides to hydrophobic ginsenosides, including heat treatment, acid hydrolysis, microwave treatment, alkaline cleavage, enzyme treatment, and fermentation. Therefore, bioconversion technology for ginseng products containing preconverted metabolites (Rg3, Rk1, Rg5, F2 (which contain two glucose molecules), and CK (which contains one glucose molecule)) is strongly considered to be useful for avoiding individual variations in ginsenoside metabolism and promoting ginsenoside absorption [21].

However, the differences in ginsenoside pharmacokinetics following RG administration in humans are still unclear. Furthermore, the pharmacokinetics of ginsenosides Rk1 and Rg5 in human plasma after oral administration remains unknown. Therefore, in this study, we aimed to elucidate the pharmacokinetic properties of PPD-type ginsenosides after oral administration of RG and bioconverted red ginseng (BRG) in healthy Korean subjects. The findings of this study provide valuable insights into the pharmacokinetics of various bioactive ginsenosides, thus contributing to enhancing the knowledge regarding the pharmacological activity of ginseng.

## 2. Materials and Methods

### 2.1. Materials and Reagents

Ginsenosides Rb1, Rg3, Rg5, Rk1, F2, and CK were purchased from Ambo Institute (Daejeon, Republic of Korea). RG (red ginseng extract C, Batch No. RG23H-190701) and BRG (FermenGIN®, Batch No. BRG FL-191014) extracts were provided by BTC Corporation (Ansan, Republic of Korea). Digoxin was used as an internal standard (IS), and formic acid was used to provide a suitable ionization environment for the analytes; both were obtained from Sigma-Aldrich Corporation (St. Louis, MO, USA). Methanol and acetonitrile (high-performance liquid chromatography (HPLC) grade) were obtained from J.T. Baker (Phillipsburg, NJ, USA). The HIQ I water purification system (Hanam, Republic of Korea) was used to prepare deionized water for HPLC analysis.

### 2.2. Preparation of Red Ginseng

Dried Korean red ginseng was purchased from Jungwon Ginseng (Geumsan, Republic of Korea). The raw dried red ginseng was extracted three times with 30 L of 50% ethanol and once with 30 L of water. The extract was then evaporated and dried using a vacuum dryer to be used as RG. To prepare BRG, RG was enzymatically treated with Sumizyme AC obtained from Shin Nihon Chemicals (Aichi, Japan) and mixed with *γ*-cyclodextrin purchased from Wacker Chemie AG (Munich, Germany) as an excipient. Subsequently, concentration and vacuum drying were performed. The prepared samples were sealed and stored at 25 ± 5°C until use in the experiments.

### 2.3. HPLC Analysis of Ginsenosides

The content of Rg1, Rb1, Rg3, Rk1 + Rg5, F2, and CK in the extracts of RG and BRG was analyzed using HPLC. Approximately 450 mg of RG and BRG samples were weighed and dissolved in 20 mL of methanol. After the samples were filtered through a nylon syringe filter, the ginsenoside content was measured using an Agilent 1200 series HPLC system with a diode-array detector (DAD; Agilent, Foster City, CA, USA). A Discovery C18 analytical column (4.0 × 250 mm, 5 *μ*m; Sigma-Aldrich, MO, USA) was used for separation. The mobile phase consisted of water (solvent A) and acetonitrile (solvent B). The flow rate of the mobile phase was 1.6 mL/min. The gradient conditions of the mobile phase were as follows: solvent A/solvent B = 80/20, 68/32, 60/40, 55/45, 25/75, 0/100, 0/100, 80/20, and 80/20, with run times of 0–10, 10–40, 40–58, 58–70, 70–93, 93–93.5, 93.5–98.5, 98.5–100, and 100–105 min, respectively. The injection volume was 10 *μ*L. The column temperature was kept constant at 25°C, and the wavelength of the UV detector was set at 204 nm.

### 2.4. Subjects

This study was approved by the Institutional Review Board of the Bestian Clinical Trial Center (approval number: BMC 2020-05-004, date of approval: May 14, 2020). All procedures of the study were carried out in compliance with the principles of the Declaration of Helsinki and Korean Good Clinical Practice guidelines. All subjects underwent screening examinations and provided written informed consent before enrolment in the study. Fourteen physically healthy Korean men aged between 20 and 45 years were enrolled. Eligibility criteria for selecting subjects included being healthy by medical history and physical examination (body mass index: 18.0–30 kg/m^2^; body weight ≥50 kg). Subjects with any significant clinical illness within 30 days before the study and blood donation within 2 weeks before the study were excluded. Subjects with alcohol abuse or those who had used any drug or food containing a large amount of saponins, such as red ginseng and ginseng, within 30 days before the study were also excluded. In addition, all subjects who experienced adverse reactions due to the ingestion of ginseng were excluded.

### 2.5. Study Design and Blood Sample Collection

**Figure 2:**
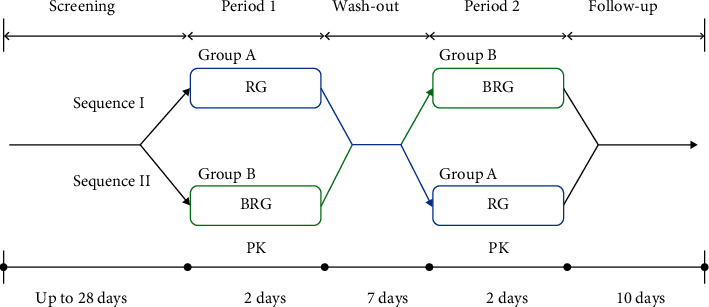
Overall study design. PK, pharmacokinetics; RG, red ginseng; BRG, bioconverted red ginseng.

This study was designed as a randomized, open-label, single-dose, crossover study with washout every 1 week to evaluate the pharmacokinetics and safety of ginsenosides. Subjects were randomly assigned to either the RG or BRG group during the first period of the study (Figure 2). After 1 week of the washout period, other products that were not received in the first period were provided. All enrolled subjects were hospitalized at Bestian Clinical Trial Center on the day before taking RG or BRG. Subjects were provided regular meals, and additional food intake was restricted after the meal. The next day, the subjects who had maintained the fasting state for 10 h took the capsules corresponding to 5 g of RG and BRG orally. Water was restricted for 1 h before and after intake, and food intake was restricted for 4 h after administration. After 4 h, all subjects received a standardized meal. In addition, lying down or sleeping was prohibited for 8 h after oral administration. Blood samples (5 mL) were collected before dosing and at each time point (0, 0.5, 1, 2, 4, 6, 8, 10, 12, and 24 h) after dosing. All blood samples were collected in heparin-treated tubes. The blood samples were promptly centrifuged at 3,000 rpm for 10 min at 4°C, and the plasma samples obtained were stored in a deep freezer at −70 ± 10°C until assay.

### 2.6. Analytic Conditions of LC-MS/MS

All analyses were performed using an Agilent 1200 series HPLC system (Agilent) with a Luna Phenyl-Hexyl column (2.0 × 150 mm, 5 *μ*m; Phenomenex, CA, USA). The mobile phase comprised water (solvent A) and acetonitrile (solvent B) containing 0.1% formic acid. The flow rate of the mobile phase was 0.25–0.35 mL/min. The initial condition of 70% solvent A was set to 5% solvent A over 15 min. The content of ginsenosides in human plasma was measured using an API 5500 mass spectrometer (AB Sciex, Foster City, CA, USA) capable of multiple reaction monitoring (MRM). The parent-daughter ion pairs monitored were 845.5 ⟶ 799.6 for Rg1, 1153.6 ⟶ 1107.5 for Rb1, 829.4 ⟶ 783.5 for Rg3, and F2, 811.4 ⟶ 161.0 for Rg5 and Rk1, 667.4 ⟶ 161.0 for CK, and 825.4 ⟶ 779.3 for digoxin as an IS in the negative ion mode. Nitrogen gas was used in the nebulizer and the collision cell. The ion spray voltage was set to 5500 V.

### 2.7. Sample Preparation

Standard plasma samples were prepared such that each ginsenoside was in the concentration range of 0.5–100 ng/mL; 10 *μ*L of a standard mixture of six kinds of ginsenosides (Rg1, Rb1, Rg5 + Rk1, F2, and CK) was added to 90 *μ*L of blank human plasma. Rg3 was prepared in the concentration range of 0.2–40 ng/mL. Standard plasma samples (100 *μ*L) were added to 200 *μ*L of methanol containing 50 ng/mL digoxin, an IS. These were mixed for 1 min and then centrifuged at 12,000 rpm for 5 min at 4°C. Then, 100 *μ*L of the supernatant was transferred to an LC vial, and 10 *μ*L was injected into the LC-MS/MS system. Plasma samples stored at −70°C were thawed at room temperature. Samples for LC-MS/MS analysis were pretreated in the same manner as standard plasma samples.

### 2.8. Method Validation

The LC-MS/MS method was validated to determine selectivity, lower limits of quantification (LLOQ), accuracy, precision, recovery, matrix effect, and stability, according to the guideline for validating the bioanalytical method [22, 23]. The selectivity of the method was evaluated by comparison of blank plasma from six different men to the corresponding spiked plasma sample at the level of LLOQ to investigate the potential interferences at the LC chromatographic conditions for ginsenoside analytes and IS. The signal-to-noise ratio (S/N) of the LLOQ sample was at least five times the response compared to the blank samples. Each calibration curve was constructed by plotting the peak area ratio (*y*) of the analyte to IS against the spiked concentrations (*x*) of each analyte. Linearity was assessed by weighted (1/*x*^2^) least squares regression analysis. The LLOQ was defined as the lowest concentration on the calibration curve with an S/N ratio of 10, where the precision and accuracy bias were within ±20% by five replicate analyses. The intra- and interday precision and accuracy assessments were performed at four concentrations with five replicates on the same day and three consecutive days, respectively. The acceptance criterion recommended by the guideline of precision and the accuracy was ±15% of the nominal concentration. The recovery of the seven ginsenosides was determined at three QC levels with three replicates by comparing the peak areas of plasma extracts spiked with analytes before extraction with those of the postextraction spiked samples at the same concentration. The matrix effects were investigated by comparing the peak areas of the analytes dissolved in the pretreated blank plasma with the corresponding concentrations prepared in the reconstitution solution with three replicates. The stability of the analytes in human plasma was assessed by analyzing five replicates of plasma samples at low, medium, and high QC levels under four different conditions. Short-term stability was evaluated after the exposure of QC samples to room temperature for 19 h before processing and analysis. The freeze and thaw stability was tested after three repeated cycles of thawing at room temperature and freezing at approximately −70°C. The long-term stability was assessed after storage at approximately −70°C for 50 days. The post-preparative stability was examined after the exposure of processed samples at ambient temperature (stored in an HPLC autosampler) for 34 h.

### 2.9. Tolerability

Side effects that might have occurred during the entire study period were monitored based on volunteer reports, questionnaires, and clinical tests. Investigators evaluated all clinical side effects in terms of severity, correlation with the drug administered, duration, and symptoms.

### 2.10. Pharmacokinetic Analysis

Plasma concentrations of seven ginsenosides were calculated from calibration curves by calculating the ratio of the peak area of the test substance to the peak area of the IS. The following pharmacokinetic parameters were measured using Phoenix WinNonlin 6.3 (Pharsight Co, CA, USA): time to reach maximum observed plasma drug concentration (*T*_max_), maximum observed plasma drug concentration (*C*_max_), area under the plasma drug concentration-time curve from 0 h to the final measured time (AUC_(0–t)_), area under the plasma drug concentration–time curve from 0 h to infinity (AUC_(0–∞)_), and terminal elimination half-life (*t*_1/2_).

### 2.11. Statistical Analysis

All data are expressed as mean ± standard deviation (SD). One-way ANOVA followed by Duncan's multiple comparisons test or Student's *t*-test was performed using the SAS statistical software (version 9.4; SAS Institute Inc., Cary, NC, USA). Differences with *p*-value <0.05 were considered significant.

## 3. Results and Discussion

### 3.1. Measurement of Ginsenoside Content in RG and BRG

Ginsenoside content in RG and BRG was calculated by substituting into the equation of the calibration curve for each compound. The ginsenoside content of each sample is shown in Table 1. The content of Rg1, Rb1, Rg3, Rg5 + Rk1, F2, and CK was 3.99, 15.51, 0.87, 1.26, 0, and 0 mg/g (not determined, ND), respectively, in RG and 0.78, 2.16, 6.07, 7.23, 2.48, and 0.56 mg/g, respectively, in BRG. Representative HPLC–DAD chromatograms of the standard mixture RG and BRG (wavelength of detection, 204 nm) are shown in Supplementary Figure S1.

### 3.2. Safety of Red Ginseng Extract for Oral Administration

**Figure 3:**
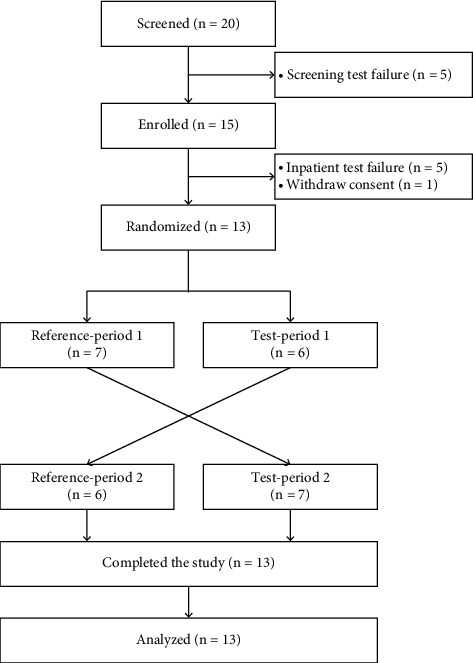
Disposition of the subjects during each step of the study.

In total, 14 healthy Korean men were enrolled, but one subject voluntarily withdrew from participation in the study (Figure 3). The mean age, height, body weight, and body mass index of the subjects were 30.62 ± 8.45 years, 173.87 ± 5.66 cm, 79.53 ± 7.84 kg, and 26.31 ± 2.09 kg/m^2^, respectively. The demographic characteristics of all subjects are shown in Supplementary Table S1. No significant differences were found in any of the characteristics between the groups.

In this study, no serious adverse reactions were observed in any subject before and after ginseng administration. On the day of hospitalization, the levels of aspartate transaminase (AST), alanine aminotransferase (ALT), and *γ*-glutamyl transpeptidase (*γ*-GTP) were measured to confirm the basal state of the subjects before administration. Blood samples were collected 24 h after dosing, and the changes in AST, ALT, and *γ*-GTP levels were confirmed through additional tests. The results of the biochemical tests in both groups are shown in Table 2. There were no clinically significant differences between the groups.

### 3.3. Validation of the Analytical Method for Measuring Plasma Ginsenoside Content

**Figure 4:**
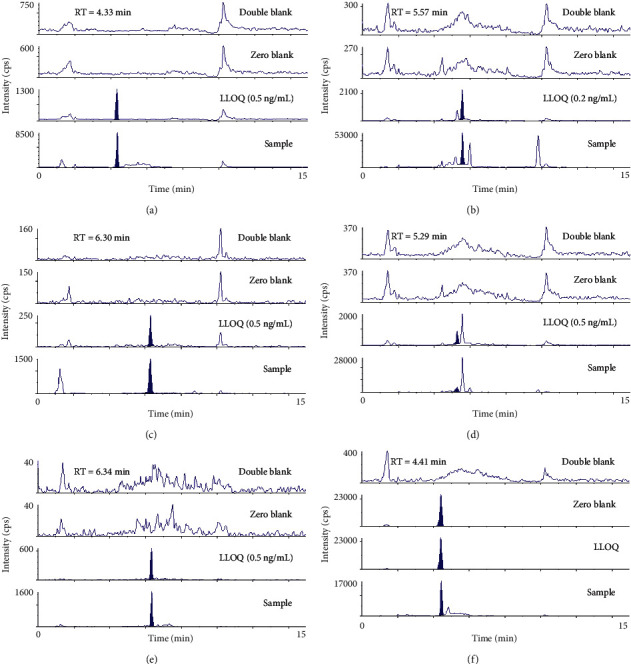
Representative multiple reaction monitoring (MRM) chromatograms of ginsenosides: (a) ginsenoside Rb1, (b) ginsenoside Rg3, (c) ginsenoside Rg5 (Rk1), (d) ginsenoside F2, (e) compound K, and (f) digoxin (IS) in human double blank plasma, blank plasma containing IS, blank plasma containing ginsenosides of LLOQ and IS, and plasma sample near *C*_max_ after a single administration of bioconverted red ginseng extract. IS, internal standard; LLOQ, lower limit of quantitation; *C*_max_, maximum concentration; RT, retention time.

Of the seven ginsenosides examined, ginsenoside Rg1 was not detected in the plasma samples. Therefore, a quantitative analysis method was set up using an LC-MS/MS system to determine the contents of the six ginsenosides in human plasma samples. Representative MRM chromatograms of the detected ginsenosides are shown in Figure 4. No endogenous interference peaks were detected in the retention time of IS and Rb1, Rg3, Rg5 + Rk1, F2, and CK. The equation of the calibration curve, correlation coefficients, LLOQ, intra- and interday precision and accuracy, matrix effect, and stability for the analysis of ginsenosides Rb1, Rg3, Rg5 + Rk1, F2, and CK were calculated. The equations of the calibration curve were *y* = 0.0814*x* + 0.0665 for Rb1; *y* = 0.2863*x* + 0.0428 for Rg3; *y* = 0.0148*x* + 0.0069 for Rg5 + Rk1; *y* = 0.0388*x* + 0.0067 for F2; *y* = 0.0301*x* + 0.0216 for CK. The correlation coefficients were 0.9991 for Rb1, 0.9998 for Rg3, 0.9997 for Rg5 + Rk1, 1.000 for F2, and 0.9996 for CK, which exhibited good linearity. The measured LLOQ were 0.5 ng/mL for Rb1, Rg5 + Rk1, F2, and CK, respectively, and 0.2 ng/mL for Rg3. These results were proven acceptable in analyzing PK behaviors of the six ginsenosides in RG or BRG (Supplementary Table S2). The intraday precision and accuracy were 1.05–5.10% and 98.94–101.60% for Rb1; 0.93–4.23% and 97.67–101.34% for Rg3; 2.59–5.26% and 97.89–102.80% for Rg5 + Rk1; 2.36–3.87% and 97.07–100.80% for F2; 2.29–3.04% and 98.37–102.80% for CK, respectively. The interday precision and accuracy were 2.95–3.98% and 99.30–100.84% for Rb1; 2.56–4.00% and 98.83–100.11% for Rg3; 2.76–5.27% and 99.80–102.16% for Rg5 + Rk1; 2.30–5.03% and 99.02–100.04% for F2; 2.09–4.28% and 98.33–101.73% for CK, respectively. It was confirmed that the intra- and interday precision and accuracy did not exceed the acceptable range (Supplementary Table S3). The extraction recovery, matrix effect, and stability are shown in Supplementary Tables S4–S6, respectively. Briefly, the mean extraction recovery of the analytes was in the range 83.66%–95.80% at different concentration levels, which indicated that the recoveries of the six analytes were consistent, precise, and reproducible in various QC samples. The matrix effects ranged within 97.04%–105.92% for the analytes. The stability of the analytes in the plasma samples had a relative standard deviation in the range of 0.67%–8.33%. These results revealed that the samples were stable throughout the PK study.

We performed the fully validated analytical method optimized for our analysis systems to fulfill the guideline for validating the bioanalytical method [22, 23]. Furthermore, the validated method was successfully used to evaluate the comparative PK phenomena of six ginsenosides of RG or BRG in Korean men.

### 3.4. Pharmacokinetics of Ginsenosides Rb1, Rg3, Rg5 + Rk1, F2, and CK after Administration

To the best of our knowledge, this is the first study to examine the pharmacokinetic characteristics of various ginsenosides such as Rg1, Rb1, Rg3, Rk1 + Rg5, F2, and CK after oral administration of RG and BRG in healthy Korean volunteers.

**Figure 5:**
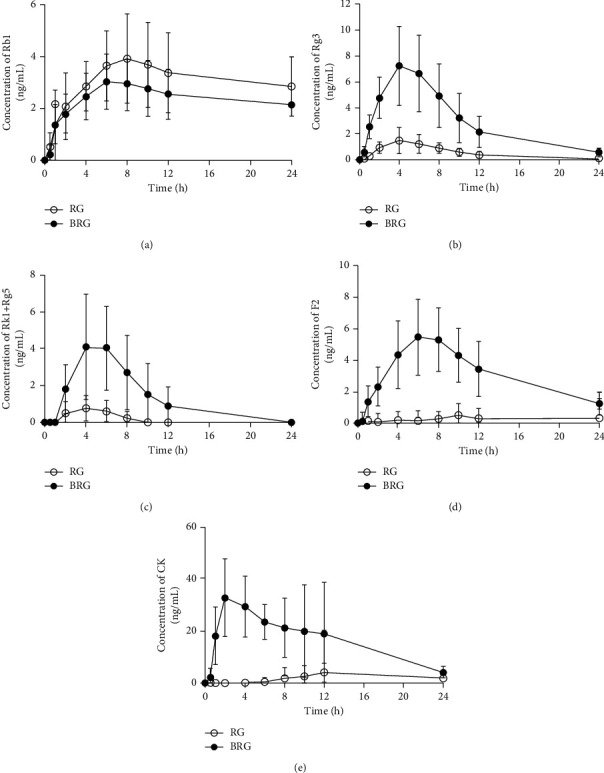
The mean ± standard deviation plasma concentration–time profiles of the ginsenosides (a) Rb1, (b) Rg3, (c) Rk1 + Rg5, (d) F2, and (e) compound K (CK) after the oral administration of RG and BRG. RG, red ginseng extract; BRG, bioconverted red ginseng extract.

The analysis method to derive the results for each pharmacokinetic parameter was utilized in the pharmacokinetic study of ginsenosides after a single dose of 5 g RG or BRG to 13 healthy Korean men. The mean plasma concentration-time curves are shown in Figure 5. At all time points, there were significant differences in the pharmacokinetic parameters between the RG and BRG groups (Table 3). Rg3, Rk1 + Rg5, F2, and CK ginsenosides showed higher *C*_max_, AUC_(0–t)_, and AUC_(0–∞)_ after oral administration of BRG compared to that after administering RG. The significant increase in the AUC and *C*_max_ of ginsenosides indicated that BRG ginsenosides were absorbed better than RG following intragastric administration, even after considering the differences in the composition ratio of ginsenosides between the two extracts. The content of ginsenoside Rk1 + Rg5 in BRG was 5.73 times higher than that of RG, and the value of AUC_(0–t)_ of ginsenoside Rk1 + Rg5 in the BRG group was approximately 7.92 times higher than that of all ginsenosides. Moreover, the BRG group showed a very low CK content but >28.0 times higher AUC_(0–t)_ values than the RG group. Interestingly, in the BRG group, the content of F2 was >4.42 times higher than that of CK (Table 1), but the value of AUC_(0–t)_ of CK was >5.03 times higher than that of F2. Furthermore, the mean *T*_max_ of CK in the BRG group was 4.77 ± 3.61 h, which indicates that the absorbed amount of CK in this group was greater than that in the RG group. Furthermore, additional analyses were performed using the Duncan post hoc test to observe the effects of group, timing, and change in subjects within the groups on AUC_(0–∞)_ and *C*_max_. The analysis revealed significant differences in Rg3, Rk1 + Rg5, F2, and CK (Supplementary Table S2). These results suggest that bioconverted ginsenosides may improve the bioavailability of CK, thus affecting its pharmacological effects.

A crossover design with randomization has a powerful advantage. The effects of RG and BRG have been compared within a subject such that each subject served as their own control. This design removes the intersubject variability and the effect of covariates that may affect the dependent variables [24].

However, this design has some limitations. A sufficient washout period is necessary until the effect of the first treatment subsides and the residues of the first treatment disappear. Therefore, if a treatment substance has an extended half-life, it is difficult to conduct a study with a crossover design. Furthermore, it is necessary to ensure that the residues of the first substance do not remain during the period of treatment with the second substance. Considering these points, we had a washout period of 1 week between the first and second treatment trials. During the first period, 6 subjects (group 1) took the test extract, and 7 subjects (group 2) took the reference extract; and in the second phase, subjects in group 2 took the test extract, and group 1 took the reference extract. Furthermore, in the results of pharmacokinetic analysis obtained during the first period and the second period, we did not detect Rg3, Rk1 + Rg5, F2, CK, and Rb1 (*t*_1/2_ = 38–46 h) in the plasma samples taken at the initial time point (0 h) of the second treatment period (Supplementary Figure S2).

Several studies have shown that there is a large difference in the metabolic activities of gut microbiota among individuals. The AUC_(0–t)_ value of CK was 14.49 ± 30.45 ng∙h/mL in the RG group, although the content of F2 was lower than the quantitative limit. The mean *T*_max_ of CK in the RG group was 8.15 ± 10.21 h, which was approximately 1.71 times lower than that in the BRG group. This indicates that other ginsenosides in the metabolic pathway, including F2, are slowly converted to CK by intestinal microbial flora. In general, the PPD-type ginsenosides Rb1, Rg2, and Rc can be biologically converted to CK, and ginsenosides Rg3, Rk1, and Rg5 can be transformed into PPD by gut microbiota. The pharmacokinetic parameters of PPD from RG in humans have not been elucidated. However, Kim et al. reported that *C*_max_ of CK was 8.35 ± 1.97 ng/mL, which was considerably higher than that of ginsenoside Rb1 (3.94 ± 1.97 ng/mL), and the half-life of CK was seven times shorter than that of Rb1 [25]. These results suggest that gut microbiota play a pivotal role in the conversion of bioactive ginsenosides. However, direct administration of BRG extract may lead to an increased absorption rate in the body, resulting in improved pharmacological efficiency and effectiveness.

## 4. Conclusions

This study revealed that ginsenosides Rg3, Rk1 + Rg5, F2, and CK showed a higher *C*_max_, AUC_(0–t)_, and AUC_(0–∞)_ and shorter *T*_max_(for CK) after BRG administration compared to those after RG administration, suggesting that BRG may lead to a higher absorption rate of bioactive ginsenosides. Thus, consuming various bioactive ginsenosides is essential to enhance the pharmacological activities of ginseng. We showed that the pharmacokinetic properties of ginsenosides, including Rb1, Rg3, Rk1 + Rg5, F2, and CK, after BRG administration may provide valuable information for future studies investigating the role of ginsenosides in the therapeutic efficacy of RG. This also indicates that the metabolism and absorption of ginsenosides in the body may be affected by the individual intestinal microbial environment. In conclusion, the conversion of ginsenosides into an easily absorbable form using bioconversion technology may increase the bioavailability of RG.

## Figures and Tables

**Table 1:** Compositional analysis of red ginseng and bioconverted red ginseng by HPLC.

Ginsenosides	Rg1 (mg/g)	Rb1 (mg/g)	Rg3 (mg/g)	Rk1 + Rg5 (mg/g)	F2 (mg/g)	CK (mg/g)	Total (mg/g)
RG	3.99	15.51	0.87	1.26	ND	ND	21.63
BRG	0.78	2.16	6.07	7.23	2.48	0.56	19.28

RG, red ginseng; BRG, bioconverted red ginseng; ND, not determined.

**Table 2:** Biochemical analysis of human plasma from healthy Korean men before and after the oral administration of red ginseng extract.

Parameter	RG			BRG			*p*-value
	Before	After	*p*-value	Before	After	*p*-value	
ALT (U/L)	23.46 ± 9.67	20.85 ± 8.49	0.024	25.77 ± 12.39	25.23 ± 12.04	0.113	0.418
AST (U/L)	24.62 ± 7.04	20.08 ± 4.21	0.002	26.23 ± 8.15	25.15 ± 16.37	0.135	0.360
*γ*-GTP (U/L)	33.69 ± 15.15	31.31 ± 13.66	0.003	33.08 ± 14.54	31.00 ± 13.69	0.008	0.935

All values are presented as mean ± standard deviation. AST, aspartate transaminase; ALT, alanine aminotransferase; *γ*-GTP, *γ*-glutamyl transpeptidase.

**Table 3:** Pharmacokinetic parameters of ginsenosides after oral administration.

Ginsenosides	Ginseng	*T*_max_ (h)	*C*_max_ (ng/mL)	AUC_(0–t)_ (ng·h/mL)	AUC_(0–∞)_ (ng·h/mL)	*T*_1/2_ (h)
Rb1	RG	7.23 ± 1.92	4.04 ± 1.71	73.25 ± 29.81	257.42 ± 115.26	45.93 ± 21.91
	BRG	6.92 ± 1.55	3.19 ± 1.13	56.93 ± 19.61	182.26 ± 94.43	38.06 ± 13.48

Rg3	RG	3.85 ± 1.28	1.51 ± 0.98	11.79 ± 7.40	14.14 ± 8.05	5.58 ± 3.63
	BRG	4.23 ± 1.54	7.70 ± 3.17^*∗*^	71.55 ± 31.05^*∗*^	76.04 ± 33.31^*∗*^	5.70 ± 0.73^*∗*^

Rk1 + Rg5	RG	3.08 ± 2.10	0.92 ± 0.71	3.54 ± 3.46	3.26 ± 5.22	1.60 ± 2.93
	BRG	5.23 ± 1.01^*∗*^	4.54 ± 2.74^*∗*^	28.06 ± 19.93^*∗*^	34.53 ± 25.10^*∗*^	2.70 ± 0.66

F2	RG	ND	ND	ND	ND	ND
	BRG	6.62 ± 1.71^*∗*^	5.83 ± 2.35^*∗*^	74.09 ± 32.97^*∗*^	90.67 ± 43.02^*∗*^	9.77 ± 7.71^*∗*^

CK	RG	8.15 ± 10.21	1.99 ± 3.27	14.49 ± 30.45	3.72 ± 7.91	1.36 ± 2.61
	BRG	4.77 ± 3.61	37.72 ± 17.78^*∗*^	406.16 ± 242.21^*∗*^	456.66 ± 258.91^*∗*^	7.72 ± 4.66^*∗*^

All values are presented as mean ± standard deviation. *T*_max_, time to reach maximum plasma concentration; *C*_max_, maximum plasma concentration; AUC_(0–t)_, area under the plasma concentration-time curve from zero to the time of the last quantifiable concentration; AUC_(0–∞)_, area under the plasma concentration-time curve from zero to infinity; *T*_1/2_, terminal half-life; *n* = 13; ^*∗*^*p* < 0.05, significant compared with the RG group according to paired *t*-test.

## Data Availability

The data used to support the findings of this study are included within the supplementary information file.

## Supplementary Materials

Supplementary Materials Supplementary Figure S1. Representative high-performance liquid chromatography–diode-array detector (HPLC–DAD) chromatograms of (a) blank, (b) standard mixture, (c) red ginseng extract (RG), and (d) bioconverted red ginseng extract (BRG) detected at 204 nm. Standard mixture contains seven ginsenosides: (i) Rg1, (ii) Rb1, (iii) F2, (iv) Rg3, (v) CK, (vi) Rk1, and (vii) Rg5. Supplementary Figure S2. Pharmacokinetic parameters of the first treatment period (A, C, E, G, I) and second treatment period (B, D, F, H, J) for ginsenosides Rb1 (A, B), Rg3 (C, D), Rk1 + Rg5 (E, F), F2 (G, H), and compound K (CK) (I, J). Supplementary Table S1. Demographic characteristics and clinical laboratory data of 13 subjects enrolled in the study. Supplementary Table S2. Regression data and LLOQs of the ginsenosides. Supplementary Table S3. Precision and accuracy for the ginsenosides in human plasma (five replicates per day for 3 days). Supplementary Table S4. Extraction recovery and matrix effect for the ginsenosides in human plasma. Supplementary Table S5. Stability of the ginsenosides in human plasma. Supplementary Table S6. One-way ANOVA test for pharmacokinetics of ginsenosides Rb1, Rg3, Rk1 + Rg5, F2, and compound K (CK) after administration.
