# Supplementary material for: Pharmacokinetics of Ginsenoside Rb1, Rg3, Rk1, Rg5, F2, and Compound K from Red Ginseng Extract in Healthy Korean Volunteers
Source: Evid Based Complement Alternat Med. 2022 Jan 24;2022:8427519. doi: 10.1155/2022/8427519 (PMC8803428; doi:10.1155/2022/8427519)
Supplement: Supplementary Materials — Supplementary Figure S1. Representative high-performance liquid chromatography–diode-array detector (HPLC–DAD) chromatograms of (a) blank, (b) standard mixture, (c) red ginseng extract (RG), and (d) bioconverted red ginseng extract (BRG) detected at 204 nm. Standard mixture contains seven ginsenosides: (i) Rg1, (ii) Rb1, (iii) F2, (iv) Rg3, (v) CK, (vi) Rk1, and (vii) Rg5. Supplementary Figure S2. Pharmacokinetic parameters of the first treatment period (A, C, E, G, I) and second treatment period (B, D, F, H, J) for ginsenosides Rb1 (A, B), Rg3 (C, D), Rk1 + Rg5 (E, F), F2 (G, H), and compound K (CK) (I, J). Supplementary Table S1. Demographic characteristics and clinical laboratory data of 13 subjects enrolled in the study. Supplementary Table S2. Regression data and LLOQs of the ginsenosides. Supplementary Table S3. Precision and accuracy for the ginsenosides in human plasma (five replicates per day for 3 days). Supplementary Table S4. Extraction recovery and matrix effect for the ginsenosides in human plasma. Supplementary Table S5. Stability of the ginsenosides in human plasma. Supplementary Table S6. One-way ANOVA test for pharmacokinetics of ginsenosides Rb1, Rg3, Rk1 + Rg5, F2, and compound K (CK) after administration. [file 8427519.f1.docx]

**Evidence-Based Complementary and Alternative Medicine Pharmacokinetics of Ginsenoside Rb1, Rg3, Rk1, Rg5, F2, and Compound K from Red Ginseng Extract in Healthy Korean Volunteers**

**Supplementary material**

**Supplementary Figure S1.** Representative high-performance liquid chromatography–diode-array detector (HPLC–DAD) chromatograms of (a) blank, (b) standard mixture, (c) red ginseng extract (RG), and (d) bioconverted red ginseng extract (BRG) detected at 204 nm. Standard mixture contains seven ginsenosides: (i) Rg1, (ii) Rb1, (iii) F2, (iv) Rg3, (v) CK, (vi) Rk1, and (vii) Rg5

**Supplementary Figure S2.** Pharmacokinetic parameters of the first treatment period (A, C, E, G, I) and second treatment period (B, D, F, H, J) for ginenosides Rb1 (A, B), Rg3 (C, D), Rk1 + Rg5 (E, F), F2 (G, H), and compound K (CK) (I, J).

**Supplementary Table S1.** Demographic characteristics and clinical laboratory data of 13 subjects enrolled in the study.

**Supplementary Table S2**. Regression data and LLOQs of the ginsenosides

**Supplementary Table S3**. Precision and accuracy for the ginsenosides in human plasma (five replicates per day for 3 days)

**Supplementary Table S4**. Extraction recovery and matrix effect for the ginsenosides in human plasma

**Supplementary Table S5**. Stability of the ginsenosides in human plasma

**Supplementary Table S6.** One-way ANOVA test for pharmacokinetics of ginsenosides Rb1, Rg3, Rk1 + Rg5, F2, and compound K (CK) after administration

**
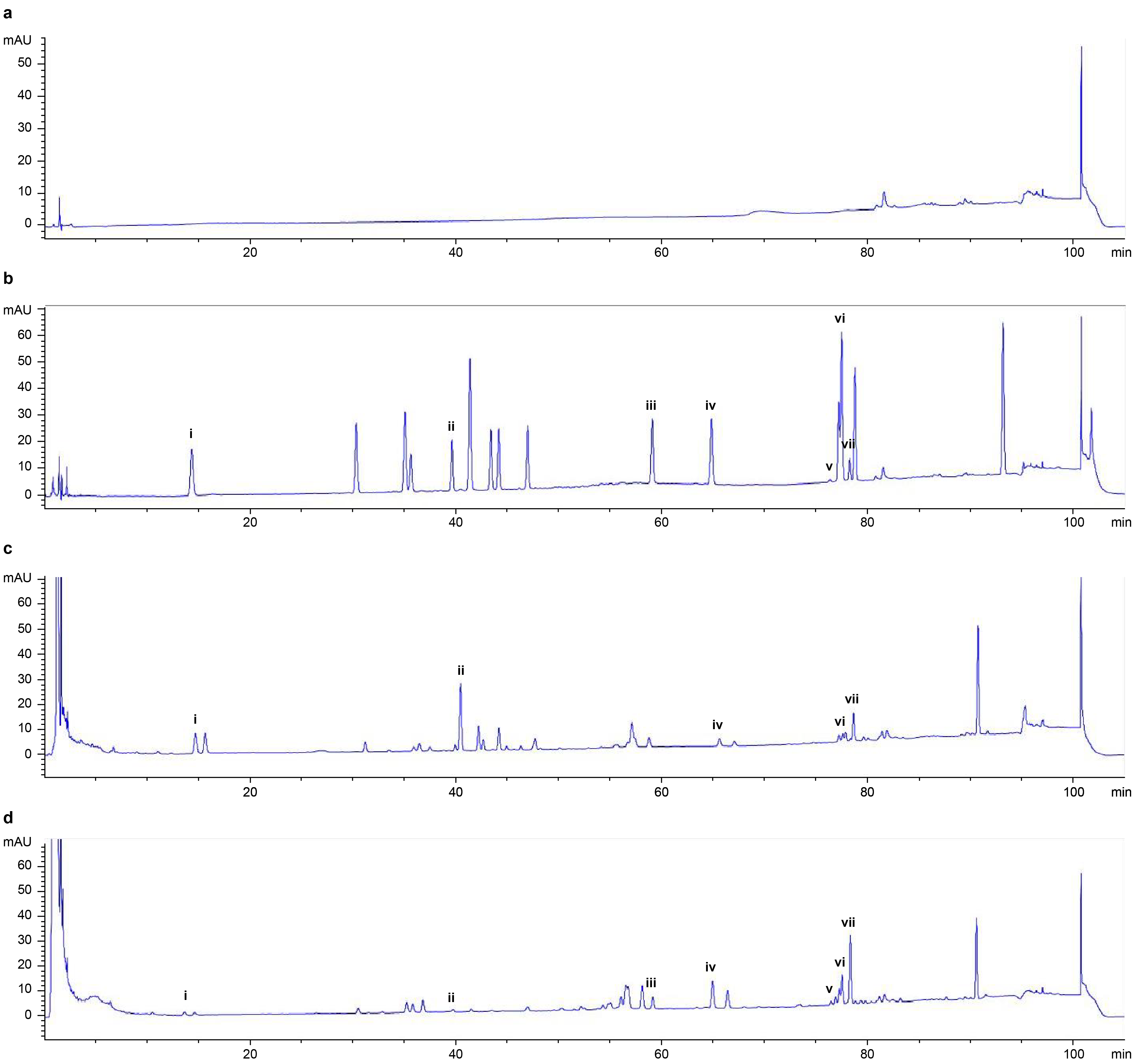
**

**Supplementary Figure S1**. Representative high-performance liquid chromatography–diode-array detector (HPLC–DAD) chromatograms of (a) blank, (b) standard mixture, (c) red ginseng extract (RG), and (d) bioconverted red ginseng extract (BRG) detected at 204 nm. Standard mixture contains seven ginsenosides: (i) Rg1, (ii) Rb1, (iii) F2, (iv) Rg3, (v) CK, (vi) Rk1, and (vii) Rg5





**Supplementary Figure S2.** Pharmacokinetic parameters of the first treatment period (A, C, E, G, I) and second treatment period (B, D, F, H, J) for ginenosides Rb1 (A, B), Rg3 (C, D), Rk1 + Rg5 (E, F), F2 (G, H), and compound K (CK) (I, J).

**Supplementary Table S1.** Demographic characteristics and clinical laboratory data of 13 subjects enrolled in the study.

| Variable | R-T Group | | T-R Group | | *p*-value |
| --- | --- | --- | --- | --- | --- |
|  | Mean | SD | Mean | SD |  |
| N | 7 | | 6 | |  |
| Age (years) | 32.43 | 9.18 | 28.50 | 7.77 | 0.4273 |
| Body height (cm) | 174.76 | 5.87 | 172.83 | 5.75 | 0.9145 |
| Body weight (kg) | 79.30 | 9.52 | 79.80 | 6.22 | 1.0000 |
| BMI (kg/m^2^) | 25.94 | 2.35 | 26.73 | 1.86 | 0.5324 |
| pH | 6.00 | 1.08 | 6.42 | 1.24 | 0.5305 |
| S.G. | 1.01 | 0.00 | 1.01 | 0.00 | 0.6991 |
| Neutrophils (%) | 52.17 | 7.95 | 52.55 | 9.97 | 0.9406 |
| Lymphocytes (%) | 36.54 | 8.45 | 36.82 | 6.44 | 0.9495 |
| Monocytes (%) | 7.97 | 2.17 | 7.52 | 2.18 | 0.7137 |
| Basophils (%) | 0.50 | 0.38 | 0.50 | 0.17 | 1.0000 |
| Eosinophils (%) | 2.81 | 2.47 | 2.62 | 1.46 | 0.8672 |
| Hb (g/dl) | 14.86 | 1.18 | 15.22 | 0.61 | 0.5157 |
| HCT (%) | 43.47 | 3.77 | 45.47 | 1.96 | 0.2691 |
| RBC (10^6^/µl) | 4.86 | 0.42 | 5.18 | 0.17 | 0.1177 |
| WBC (10^3^/µl) | 5.43 | 1.69 | 6.97 | 2.32 | 0.1934 |
| Platelet (10^3^/µl) | 245.00 | 41.92 | 235.83 | 52.30 | 0.7321 |
| Total Bilirubin (mg/dl) | 0.99 | 0.35 | 0.73 | 0.27 | 0.1759 |
| Total Protein (g/dl) | 7.33 | 0.26 | 7.30 | 0.50 | 0.8964 |
| ALT (SGPT) (IU/L) | 28.71 | 9.86 | 24.50 | 12.94 | 0.5188 |
| AST (SGOT) (IU/L) | 26.71 | 6.40 | 26.00 | 9.03 | 0.8707 |
| ALP (IU/L) | 205.00 | 28.82 | 248.33 | 77.67 | 0.2424 |
| Albumin (g/dl) | 4.53 | 0.15 | 4.62 | 0.28 | 0.4827 |
| γ-GTP (IU/L) | 47.57 | 17.47 | 25.33 | 11.43 | 0.0222 |
| Creatinine (mg/dl) | 1.11 | 0.12 | 1.02 | 0.08 | 0.1168 |
| BUN (mg/dl) | 11.73 | 3.19 | 12.22 | 2.85 | 0.7785 |
| Glucose (FBS/PP2hrs) (mg/l) | 100.43 | 5.68 | 97.00 | 6.16 | 0.3191 |
| Total Cholesterol (mg/dl) | 202.86 | 30.28 | 185.50 | 36.99 | 0.3716 |
| Systolic blood pressure (mmHg) | 130.57 | 8.89 | 125.83 | 5.34 | 0.2795 |
| Diastolic blood pressure (mmHg) | 79.86 | 10.17 | 74.83 | 5.15 | 0.2986 |
| Pulse | 70.57 | 6.08 | 67.17 | 8.42 | 0.4159 |
| Body temperature (℃) | 36.39 | 0.39 | 36.40 | 0.51 | 0.9553 |

**Abbreviations:** SD, standard deviation; BMI, body mass index; S.G., specific gravity; Hb, hemoglobin; HCT, hematocrit; RBC, red blood cell; WBC, white blood cell; ALT, alanine aminotransferase; SGPT, serum glutamic pyruvic transaminase; AST, aspartate transaminase; SGOT, serum glutamic oxaloacetic transaminase; ALP, alkaline phosphatase; γ-GTP, γ-glutamyl transpeptidase; BUN, blood urea nitrogen; FBS, fetal bovine serum; PP2hrs, postprandial blood sugar 2 hours;

**Supplementary Table S2**. Regression data and LLOQs of the ginsenosides

|  | LLOQ (ng/mL) | Linear range (ng/mL) | Linear regression equation | R^2^ |
| --- | --- | --- | --- | --- |
| Ginsenoside Rb1 | 0.5 | 0.5 – 100 | y = 0.0814x + 0.0665 | 0.9991 |
| Ginsenoside Rg3 | 0.2 | 0.2 – 40 | y = 0.2863x + 0.0428 | 0.9998 |
| Ginsenoside Rk1 + Rg5 | 0.5 | 0.5 – 100 | y = 0.0148x + 0.0069 | 0.9997 |
| Ginsenoside F2 | 0.5 | 0.5 – 100 | y = 0.0388x+ 0.0067 | 1.0000 |
| Ginsenoside CK | 0.5 | 0.5 – 100 | y = 0.0301x + 0.0216 | 0.9996 |

LLOQ, lower limit of quantification; R^2^, coefficient of determination

**Supplementary Table S3**. Precision and accuracy for the ginsenosides in human plasma (five replicates per day for 3 days)

| Analyte | concentration (ng/mL) | Intra-day | | | Inter-day | | |
| --- | --- | --- | --- | --- | --- | --- | --- |
|  |  | Mean ± SD | Precision (%) | Accuracy (%) | Mean ± SD | Precision (%) | Accuracy (%) |
| Ginsenoside Rb1 | 0.5 | 0.51 ± 0.03 | 5.10 | 101.60 | 0.50 ± 0.02 | 3.98 | 100.27 |
|  | 1.5 | 1.51 ± 0.06 | 3.78 | 100.67 | 1.51 ± 0.05 | 3.46 | 100.84 |
|  | 20 | 19.79 ± 0.21 | 1.05 | 98.94 | 20.00 ± 0.69 | 3.44 | 99.99 |
|  | 80 | 79.27 ± 1.81 | 2.28 | 99.09 | 79.44 ± 2.34 | 2.95 | 99.30 |
| Ginsenoside Rg3 | 0.2 | 0.20 ± 0.01 | 4.23 | 99.00 | 0.20 ± 0.01 | 3.41 | 99.00 |
|  | 0.6 | 0.59 ± 0.01 | 0.93 | 97.67 | 0.60 ± 0.02 | 4.00 | 100.11 |
|  | 8 | 8.10 ± 0.17 | 2.10 | 101.28 | 7.91 ± 0.20 | 2.56 | 98.83 |
|  | 32 | 32.43 ± 0.96 | 2.95 | 101.34 | 31.74 ± 0.82 | 2.58 | 99.18 |
| Ginsenoside  Rk1 + Rg5 | 0.5 | 0.51 ± 0.03 | 5.26 | 102.80 | 0.51 ± 0.03 | 5.27 | 101.87 |
|  | 1.5 | 1.48 ± 0.04 | 2.96 | 98.53 | 1.52 ± 0.05 | 3.37 | 101.16 |
|  | 20 | 19.90 ± 0.52 | 2.59 | 99.50 | 20.43 ± 0.84 | 4.12 | 102.16 |
|  | 80 | 78.31 ± 2.34 | 2.98 | 97.89 | 79.84 ± 2.20 | 2.76 | 99.80 |
| Ginsenoside F2 | 0.5 | 0.50 ± 0.02 | 3.87 | 100.80 | 0.50 ± 0.02 | 5.03 | 99.33 |
|  | 1.5 | 1.46 ± 0.04 | 2.50 | 97.07 | 1.49 ± 0.05 | 3.14 | 99.02 |
|  | 20 | 19.96 ± 0.67 | 3.36 | 99.80 | 20.01 ± 0.53 | 2.67 | 100.04 |
|  | 80 | 79.84 ± 1.88 | 2.36 | 99.80 | 79.35 ± 1.82 | 2.30 | 99.19 |
| Ginsenoside CK | 0.5 | 0.51 ± 0.01 | 2.61 | 102.80 | 0.50 ± 0.02 | 4.28 | 100.53 |
|  | 1.5 | 1.54 ± 0.04 | 2.55 | 102.40 | 1.53 ± 0.04 | 2.94 | 101.73 |
|  | 20 | 19.67 ± 0.60 | 3.04 | 98.37 | 20.07 ± 0.67 | 3.36 | 100.34 |
|  | 80 | 79.99 ± 1.83 | 2.29 | 99.99 | 78.67 ± 1.65 | 2.09 | 98.33 |

SD, standard deviation

**Supplementary Table S4**. Extraction recovery and matrix effect for the ginsenosides in human plasma

|  | concentration (ng/mL) | Extraction recovery (n =3) | | Matrix effect (n =6) | |
| --- | --- | --- | --- | --- | --- |
|  |  | Mean ± SD | RSD (%) | Mean ± SD | RSD (%) |
| Ginsenoside Rb1 | 1.5 | 90.02 ± 2.16 | 2.39 | 98.86 ± 0.01 | 0.01 |
|  | 20 | 87.61 ± 20.11 | 22.96 | – | – |
|  | 80 | 85.78 ± 2.32 | 2.70 | 97.04 ± 0.45 | 0.47 |
| Ginsenoside Rg3 | 0.6 | 87.02 ± 0.43 | 0.49 | 102.29 ± 0.01 | 0.01 |
|  | 8 | 85.11 ± 0.59 | 0.70 | – | – |
|  | 32 | 85.56 ± 0.45 | 0.53 | 100.88 ± 0.17 | 0.17 |
| Ginsenoside  Rk1 + Rg5 | 1.5 | 95.60 ± 0.93 | 0.97 | 101.54 ± 8.92 | 0.00 |
|  | 20 | 83.66 ± 1.12 | 1.34 | – | – |
|  | 80 | 87.61 ± 1.57 | 1.79 | 99.19 ± 1.82 | 0.02 |
| Ginsenoside F2 | 1.5 | 90.39 ± 1.91 | 2.11 | 105.92 ± 0.00 | 0.00 |
|  | 20 | 95.80 ± 2.33 | 2.72 | – | – |
|  | 80 | 86.78 ± 0.94 | 1.09 | 102.09 ± 0.06 | 0.06 |
| Ginsenoside CK | 1.5 | 93.03 ± 12.36 | 13.29 | 99.73 ± 0.00 | 0.00 |
|  | 20 | 85.07 ± 0.54 | 0.64 | – | – |
|  | 80 | 87.58 ± 1.82 | 2.07 | 102.15 ± 0.04 | 0.04 |

SD, standard deviation; RSD, relative standard deviation

**Supplementary Table S5**. Stability of the ginsenosides in human plasma

| Analyte | concentration (ng/mL) | Short-term stability  (19 h at room temperature) | | Freeze-thaw stability  (3 cycles) | | Autosampler stability  (34 h at ambient temperature) | | Long-term stability  (50 days at -70℃) | |
| --- | --- | --- | --- | --- | --- | --- | --- | --- | --- |
|  |  | Concentration found (%) | RSD(%) | Concentration found (%) | RSD(%) | Concentration found (%) | RSD(%) | Concentration found (%) | RSD(%) |
|  |  | Mean ± SD |  | Mean ± SD |  | Mean ± SD |  | Mean ± SD |  |
| Rb1 | 1.5 | 98.19 ± 1.40 | 1.43 | 98.65 ± 0.66 | 0.67 | 97.98 ± 3.55 | 3.48 | 102.01 ± 1.78 | 1.74 |
|  | 80 | 96.93 ± 5.41 | 5.58 | 100.70 ± 2.36 | 2.35 | 100.35 ± 5.32 | 5.34 | 105.06 ± 2.73 | 2.59 |
| Rg3 | 0.6 | 102.32 ± 2.68 | 2.62 | 97.73 ± 3.53 | 3.61 | 102.95 ± 5.52 | 5.36 | 104.15 ± 8.68 | 8.33 |
|  | 32 | 96.32 ± 1.48 | 1.53 | 95.33 ± 2.75 | 2.88 | 97.25 ± 1.45 | 1.49 | 99.84 ± 3.12 | 3.13 |
| Rk1 + Rg5 | 1.5 | 98.50 ± 4.37 | 4.44 | 100.48 ± 1.97 | 1.96 | 99.12 ± 3.60 | 3.63 | 99.13 ± 2.12 | 2.14 |
|  | 80 | 103.11 ± 3.61 | 3.50 | 104.28 ± 2.08 | 2.00 | 102.90 ± 1.68 | 1.63 | 101.41 ± 2.51 | 2.47 |
| F2 | 1.5 | 99.21 ± 7.70 | 7.76 | 98.29 ± 4.06 | 4.13 | 99.02 ± 8.01 | 8.09 | 101.83 ± 3.76 | 3.69 |
|  | 80 | 96.53 ± 4.04 | 4.18 | 95.95 ± 1.10 | 1.14 | 96.29 ± 0.99 | 1.03 | 99.02 ± 2.04 | 2.06 |
| CK | 1.5 | 99.90 ± 4.38 | 4.39 | 104.03 ± 7.57 | 7.27 | 101.24 ± 4.59 | 4.54 | 103.25 ± 4.03 | 3.90 |
|  | 80 | 98.69 ± 2.03 | 2.05 | 96.17 ± 2.51 | 2.61 | 99.56 ± 1.44 | 1.45 | 103.02 ± 5.53 | 5.37 |

SD, standard deviation; RSD, relative standard deviation

**Supplementary Table S6.** One-way ANOVA test for pharmacokinetics of ginsenosides Rb1, Rg3, Rk1 + Rg5, F2, and compound K (CK) after administration

| Parameter | Source | AUC_(0–∞)_ (ng·h/mL) | | | | | C_max_ (ng/mL) | | | | |
| --- | --- | --- | --- | --- | --- | --- | --- | --- | --- | --- | --- |
|  |  | SS | df | MS | F | *p*-value | SS | df | MS | F | *p*-value |
| Rb1 | Between groups | 4491.643 | 3 | 1497.214 | 2.631 | 0.075 | 11.933 | 3 | 3.978 | 2.020 | 0.140 |
|  | Within groups | 12520.767 | 22 | 569.126 |  |  | 43.310 | 22 | 1.969 |  |  |
|  | Total | 17012.410 | 25 |  |  |  | 55.243 | 25 |  |  |  |
| Rg3 | Between groups | 23430.800 | 3 | 7810.267 | 14.309 | <0.001 | 251.068 | 3 | 83.689 | 14.135 | <0.001 |
|  | Within groups | 12008.376 | 22 | 545.835 |  |  | 130.253 | 22 | 5.921 |  |  |
|  | Total | 35439.176 | 25 |  |  |  | 381.321 | 25 |  |  |  |
| Rk1 + Rg5 | Between groups | 3999.874 | 3 | 1333.291 | 6.085 | <0.01 | 91.760 | 3 | 30.587 | 7.485 | <0.001 |
|  | Within groups | 4820.797 | 22 | 219.127 |  |  | 89.905 | 22 | 4.087 |  |  |
|  | Total | 8820.671 | 25 |  |  |  | 181.665 | 25 |  |  |  |
| F2 | Between groups | 36015.691 | 3 | 12005.230 | 20.781 | <0.001 | 222.204 | 3 | 74.068 | 25.181 | <0.001 |
|  | Within groups | 12709.247 | 22 | 577.693 |  |  | 64.710 | 22 | 2.941 |  |  |
|  | Total | 48724.938 | 25 |  |  |  | 286.915 | 25 |  |  |  |
| CK | Between groups | 1132614.837 | 3 | 377538.279 | 14.328 | <0.001 | 9255.940 | 3 | 3085.313 | 22.896 | <0.001 |
|  | Within groups | 579678.669 | 22 | 26349.030 |  |  | 2964.543 | 22 | 134.752 |  |  |
|  | Total | 1712293.506 | 25 |  |  |  | 12220.484 | 25 |  |  |  |

SS, Sum of squares; df, Degrees of freedom; MS, Mean square
